# Exaptive origins of regulated mRNA decay in eukaryotes

**DOI:** 10.1002/bies.201600100

**Published:** 2016-07-20

**Authors:** Fursham M. Hamid, Eugene V. Makeyev

**Affiliations:** ^1^ School of Biological SciencesNanyang Technological UniversitySingaporeSingapore; ^2^ Centre for Developmental NeurobiologyKing's College LondonLondonUK

**Keywords:** antiviral defense, exaptation, mRNA decay, nonsense‐mediated decay, regnase, roquin, tristetraprolin

## Abstract

Eukaryotic gene expression is extensively controlled at the level of mRNA stability and the mechanisms underlying this regulation are markedly different from their archaeal and bacterial counterparts. We propose that two such mechanisms, nonsense‐mediated decay (NMD) and motif‐specific transcript destabilization by CCCH‐type zinc finger RNA‐binding proteins, originated as a part of cellular defense against RNA pathogens. These branches of the mRNA turnover pathway might have been used by primeval eukaryotes alongside RNA interference to distinguish their own messages from those of RNA viruses and retrotransposable elements. We further hypothesize that the subsequent advent of “professional” innate and adaptive immunity systems allowed NMD and the motif‐triggered mechanisms to be efficiently repurposed for regulation of endogenous cellular transcripts. This scenario explains the rapid emergence of archetypical mRNA destabilization pathways in eukaryotes and argues that other aspects of post‐transcriptional gene regulation in this lineage might have been derived through a similar exaptation route.

**Abbreviations**
(T)ZF: (tandem) zinc finger
ARE: AU‐rich RNA element
EJC: exon junction complex
LECA: last common eukaryotic ancestor
NMD: nonsense‐mediated decay
ORF: open reading frame
PRR: pattern recognition receptor
RBP: RNA‐binding protein

## Introduction

Extensive regulation of gene expression at the post‐transcriptional level is a characteristic trait of eukaryotic biology differentiating this domain of life from bacteria and archaea. A compelling example of this divergence is provided by mRNA destabilization mechanisms triggered by specific nucleotide and structural elements, base‐pairing between RNA molecules and unusual patterns of mRNA translation 1, 2, 3.

When discussing mRNA decay it is important to distinguish between the core machinery responsible for RNA degradation catalysis and controlled mechanisms that target specific subsets of mRNAs. In eukaryotes, the core machinery contains the Xrn family 5′–3′ exoribonucleases and the RNA exosome, a molecular complex combining 3′–5′ exoribonuclease and endoribonuclease activities 4, 5, 6. Eukaryotic mRNA decay is typically initiated by shortening of the 3′ poly(A) tail and subsequent removal of the 5′ cap structure by deadenylation and decapping enzymes, respectively 6, 7.

Prokaryotes degrade their mRNAs using a distinct set of endo‐ and 5′–3′ exoriboucleases 4, 8, 9. Prokaryotic mRNAs are not capped but contain a 5′‐terminal triphosphate, which is removed by a pyrophosphohydrolase to facilitate subsequent degradation steps 8, 9. Degradation of prokaryotic mRNAs in the 3′–5′ direction is catalyzed by either a homomeric PNPase (bacteria) or a heteromeric exosome complex (archea) distantly related to its eukaryotic counterpart 4, 8, 9.

The difference between controlled mechanisms of mRNA decay in the two groups is arguably even more striking. In eukaryotes, the regulation is provided by small interfering (si), micro (mi) and Piwi‐interacting (pi) RNAs, nonsense‐mediated decay (NMD), and RNA‐binding protein‐based mechanisms targeting mRNAs with characteristic sequence or secondary structure elements for degradation 10, 11, 12, 13. Combined with the widespread recruitment of mRNAs to ribonucleoprotein complexes 14, these mechanisms underlie the remarkable variability in eukaryotic mRNA half‐lives ranging from minutes to days.

Prokaryotic logic of controlled mRNA decay is fundamentally different 8, 9, 15. The lack of nucleocytoplasmic compartmentalization allows bacterial and archaeal mRNAs to be translated in a co‐transcriptional manner. This is typically followed by rapid clearance of full‐length mRNAs by the core degradation machinery. A few RNA‐binding proteins (RBPs) are known to modulate mRNA stability in prokaryotes but the scope of this regulation is substantially more limited compared to eukaryotic RBPs 12, 16. Nonetheless, there is also evidence for transcript‐specific destabilization mechanisms in prokaryotes. Some of these rely on folded RNA elements acting either in cis or in trans 8, 9, 17, 18. One of the most selective mechanisms of mRNA decay in prokaryotes is provided by some types‐III and ‐VI CRISPR‐Cas immunity systems 19, 20, 21, 22, 23. In this case, CRISPR RNAs guide sequence‐specific degradation of pathogen‐derived RNAs. Importantly, these defense systems are prokaryotic inventions phylogenetically unrelated to eukaryotic RNA silencing mechanisms.

Assuming that eukaryotes originated through symbiosis between archaea and bacteria and inherited a prokaryotic version of mRNA metabolism 24, a pertinent question is why their mRNA degradation mechanisms diverged so markedly from the prokaryotic roots. A commonly held view is that segregation of transcription and translation between the nucleus and the cytoplasm provided eukaryotes with a unique opportunity to elaborate their post‐transcriptional regulation in the course of evolution 25. However, most post‐transcriptional mechanisms including those controlling mRNA destabilization are remarkably conserved across a wide range of eukaryotic organisms. This argues that eukaryotes might have acquired a bulk of their distinctive post‐transcriptional pathways prior to radiation of the major supergroups from the last common eukaryotic ancestor (LECA) and subsequently explored possibilities available within a largely established mechanistic framework.

What could have triggered rapid rewiring of the mRNA metabolism in the newly established eukaryotic domain? Here we propose that at least some of the mechanisms regulating eukaryotic mRNA stability might have emerged as a part of cellular defense against RNA pathogens. According to this scenario, subsequent appearance of specialized innate and adaptive immunity systems allowed the host cells to repurpose, or “exapt” 26, these primeval defense mechanisms for endogenous gene regulation functions.

## Eukaryotes have been exposed to a wide range of RNA pathogens throughout their evolutionary history

Viruses are exceptionally successful pathogens using their cellular hosts as a source of translation machinery, nucleotides and, occasionally, structural molecules and enzymes 27, 28, 29, 30. Life cycles of most viruses include a viral particle, or virion, facilitating the spread of the infection between cells and an intracellular stage used for replication. Depending on the type of nucleic acid contained in the virion and replication mechanisms, viruses are classified into DNA viruses with double‐ and single‐stranded genomes and RNA viruses with double‐stranded, positive single‐stranded (+), and negative single‐stranded (−) genomes. Two additional groups containing RNA or DNA genomes reverse‐transcribe RNA as a part of their replication cycles and are collectively referred to as retroid viruses. RNA is also used for replication of virus‐like entities including retrotransposable elements and viroids.

Although RNA‐dependent viruses have been isolated from both eukaryotic and prokaryotic hosts, an overwhelming majority of these pathogens infects eukaryotes 28. The current ICTV classification (http://www.ictvonline.org/virusTaxonomy.asp; 2014 Release) lists 523 prokaryotic and 2666 eukaryotic viruses. Although not an exhaustive catalog of all known species, this sizeable sample suggests that RNA viruses, retroid viruses, and viroids account for ∼62% of eukaryotic viral species, whereas the corresponding figure for prokaryotes is only ∼1%. This striking over‐representation of eukaryotic RNA pathogens does not take into account eukaryotic retroelements, which appear more diverse than such elements in bacteria 28, 31, 32, 33, 34.

Earlier phylogenetic analyses suggested that positive‐strand RNA viruses sharing picorna‐like genome architecture emerged through a “Big‐Bang” event preceding radiation of the major eukaryotic supergroups 35. This type of viruses may have evolved through recombination events involving a reverse transcriptase from a bacterial group‐II self‐splicing intron and several other components originating from the protobacterial predecessor of eukaryotic mitochondria 35. Other lineages of eukaryotic RNA‐dependent viruses might have branched off the picorna‐like tree or evolved from the two known families of prokaryotic RNA viruses, *Leviviridae* and *Cystoviridae* 28.

Eukaryotic retroid viruses and retrotransposable elements likely descended from prokaryotic retroelements 28. It has been proposed that prokaryotic retrotransposable group‐II introns additionally gave rise to eukaryotic spliceosomal introns and parts of the spliceosomal complexes 36, 37. According to different models, group‐II introns originating from the proteobacterial endosymbiont colonized the host genome either before or after the emergence of the nuclear envelope 37, 38.

## Efficient defense against RNA pathogens may require multitier immunity

Regardless of the exact evolutionary trajectories followed by specific groups of viruses and retroelements, it is fair to assume that eukaryotes have been exposed to an increasingly wide range of RNA‐based pathogens since their early days. If so, long‐term survival of the newly established eukaryotic lineage would have been impossible without adequate defense mechanisms.

**Figure 1:**
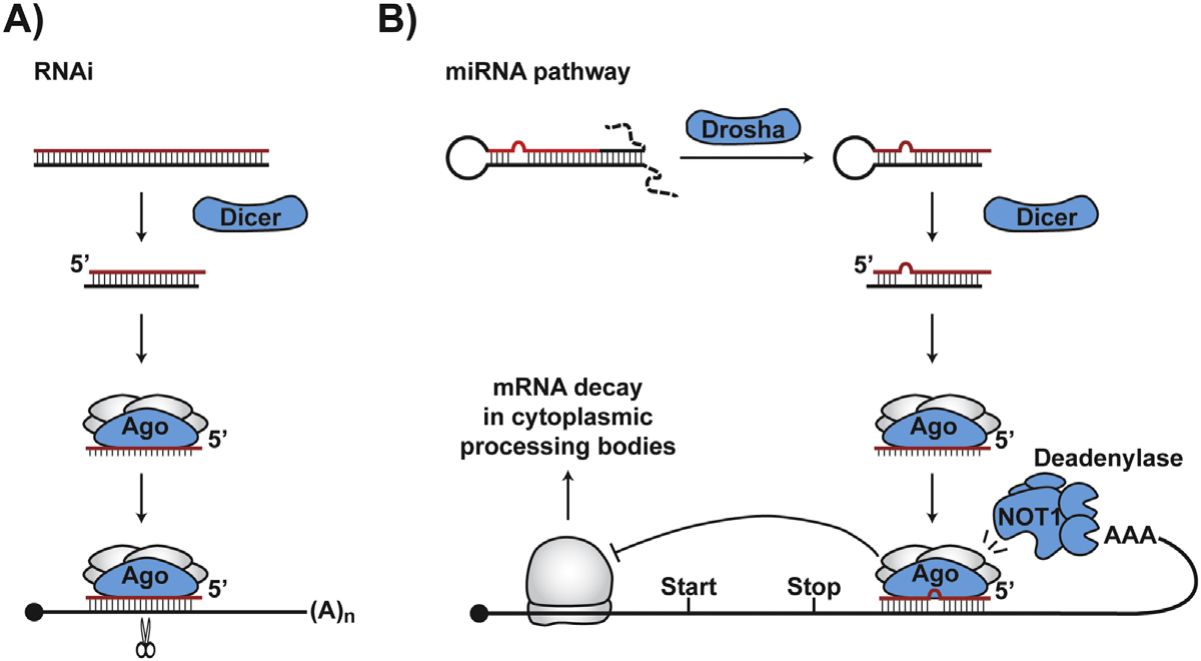
A general outline of Dicer‐ and Argonaute‐dependent post‐transcriptional RNA silencing mechanisms in eukaryotes. **A:** RNA interference (RNAi) pathway relying on Dicer‐dependent fragmentation of long dsRNA triggers into siRNA guides and Argonaute‐dependent cleavage on mRNAs complementary to the siRNAs. This simplified diagram does not show RdRP‐dependent steps amplifying RNAi response in some species. **B:** miRNA pathway related to RNAi and using partially overlapping or paralogous RNA processing enzymes to silence eukaryotic genes at the level of mRNA translation and/or stability.

One such mechanism present in most extant eukaryotes and likely used by the LECA is RNA interference (RNAi) 10, 39, 40, 41, 42, 43, 44, 45 (Fig. 1A). RNAi is triggered by dsRNAs, which are rare in eukaryotic cells but commonly produced during RNA virus replication or as a result of repeated retrotransposition events. Processing of dsRNAs by the Dicer family endoribonucleases generates ∼21‐25‐nt siRNA products guiding the Argonaute family endoribonucleases to complementary RNA targets. In addition to Dicers and Argonautes, some eukaryotes encode cellular RNA‐dependent RNA polymerases (RdRPs) that can amplify the RNAi response by synthesizing secondary dsRNA triggers or siRNAs.

Metazoans use several additional levels of antiviral defense including the Piwi/piRNA system targeting retrotransposons, innate immunity based on recognition of pathogen‐associated molecular patterns (PAMPs) by host‐encoded pattern recognition receptors (PRRs), and immunoglobulin‐ and T‐cell receptor‐dependent adaptive immunity 46, 47, 48, 49, 50, 51, 52, 53, 54, 55. Similarly, plants have developed sophisticated innate immunity mechanisms that are largely unrelated to their metazoan counterparts 53, 56, 57, 58, 59. These examples argue that robust protection against pathogens may require several lines of defense. Following this logic, lasting biosafety of ancestral eukaryotes might have required additional mechanisms re‐enforcing RNAi‐based immunity and capable of discriminating between non‐infectious “self” and infectious “nonself” 48. It is possible that such primeval mechanism(s) working alongside RNAi at early stages of eukaryotic evolution were repurposed for cellular gene regulation and eventually lost their status of a dedicated defense system.

RNAi itself has important functions unrelated to immunity and might completely forego its antiviral responsibilities in many mammalian cells 52, 60. Similarly, the miRNA pathway sharing common evolutionary roots with RNAi appears to be an example of functional exaptation. miRNAs are short single‐stranded molecules produced in metazoans and plants from endogenously encoded stem‐loop precursors 10, 41, 61 (Fig. 1B). miRNA biogenesis is catalyzed by Dicer‐family endonucleases also involved in the RNAi pathway. Mature miRNAs function as sequence‐specific guides directing repressive Argonaute‐containing complexes to their mRNA targets. This affords global regulation of cellular gene expression at the level of mRNA stability and translational efficiency.

The animal and plant miRNA pathways appear to have evolved independently following radiation of the corresponding supergroups, Opisthokonta and Archaeplastida, from a common ancestor 40. According to a popular model, miRNAs originated as retrotransposon‐derived and retrotransposon‐targeting branches of the RNAi pathway but were subsequently repurposed for regulation of cell‐specific genes 52, 62, 63. The emergence of the piRNA/Piwi pathway in metazoans and diversification of small RNA biogenesis mechanisms in plants could have facilitated this process 41, 46, 47.

Below, we discuss the possibility that, similar to RNAi and the miRNA pathway, two well‐characterized eukaryotic systems regulating stability of cell‐encoded mRNAs, emerged as intrinsic defense mechanisms against RNA pathogens.

## NMD controls mRNA quality based on translation termination patterns

**Figure 2:**
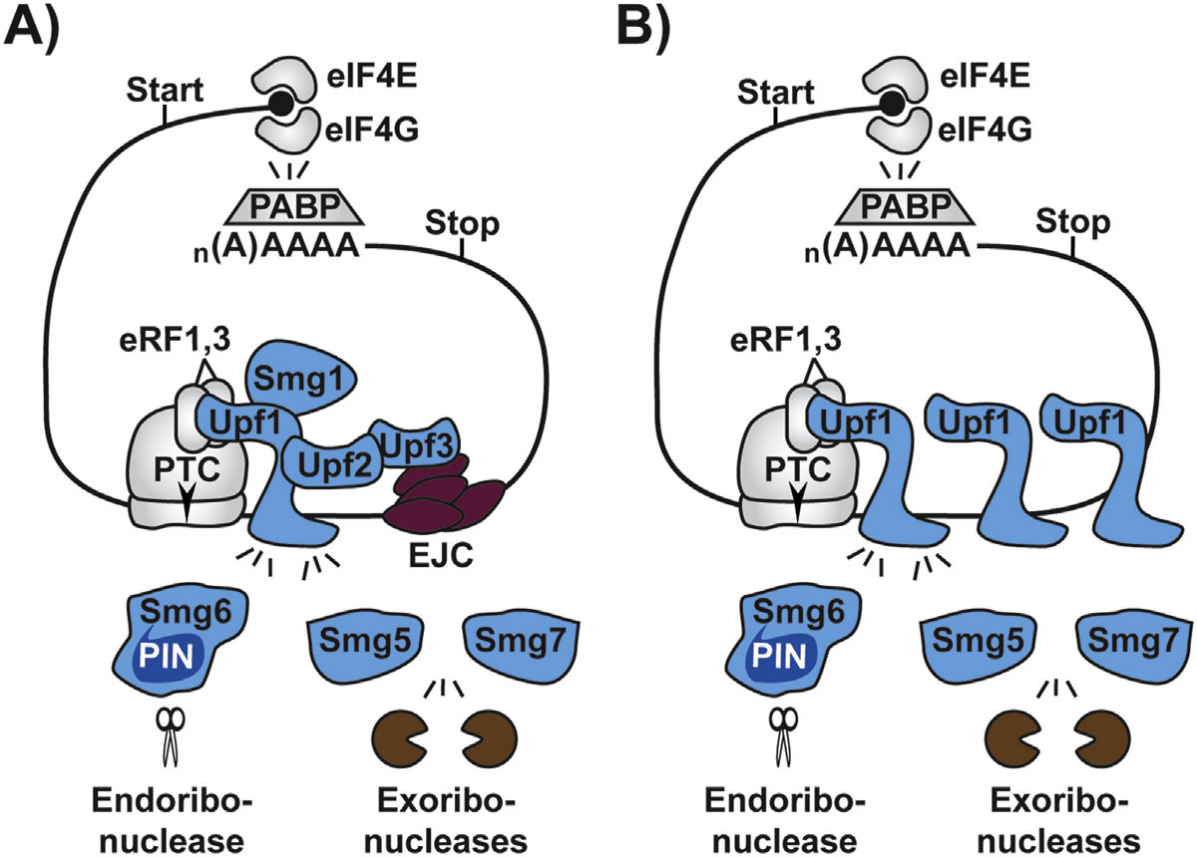
A general outline of mammalian NMD including (A) EJC‐dependent mechanism and (B) EJC‐independent mechanism. Note that the exact composition of the NMD machinery may differ depending on eukaryotic group. Abbreviations: eIF, eukaryotic initiation factor; EJC, exon junction complex; PABP, poly(A) binding protein; PTC, premature termination codon.

NMD is a conserved eukaryotic mechanism destabilizing mRNAs with unusually positioned translation stop codons 11, 64, 65, 66, 67, 68, 69, 70, 71 (Fig. 2). NMD has been originally shown to target aberrant mRNA species acquiring a premature termination codon (PTC) as a result of mutation or splicing errors. However, it is becoming increasingly clear that, in addition to this error surveillance function, NMD can control gene expression in many normal situations ranging from maintenance of RBP homeostasis to cellular differentiation and stress response.

As discussed in several recent reviews 11, 64, 65, 66, 67, 68, 69, 70, 71, the NMD machinery typically comprises the key helicase Upf1, its partners Upf2 and Upf3 and at least one member of the Smg5‐Smg6‐Smg7 group of proteins recruited to activated Upf1 and mediating target mRNA degradation. In many species, the NMD machinery also includes the Upf1 kinase Smg1 and the multisubunit exon junction complex (EJC). These components dynamically interact with several additional factors including cap‐binding proteins, translation termination factors, and mRNA degradation enzymes.

mRNAs are typically recognized as NMD targets in the cytoplasm during initial rounds of translation. The presence of exon‐exon junctions >50–55 nt downstream of the termination codon, upstream ORFs in the 5′UTR, or an excessively long 3′UTR increases the likelihood of an mRNA to undergo NMD. Mechanistic details of this pathway differ depending on the species, and a single organism may utilize more than one distinct branch of NMD (Fig. 2). For example, Upf1 appears to use several distinct mechanisms to associate with its mRNA targets. In many cases, it is recruited to PTC‐containing transcripts in an EJC‐dependent manner through the Upf2 and/or the Upf3 adapters. This is facilitated by direct interaction between Upf3 and the EJC. However, Upf1 can also interact with mRNAs in the absence of EJC with a relatively relaxed sequence specificity thus increasing the likelihood of transcripts containing long 3′UTRs to enter the NMD pathway. This mechanism can induce NMD, for example, in transcripts of intron‐less genes and is especially widespread in intron‐poor species such as *Saccharomyces cerevisiae*.

Notwithstanding this functional diversity, genes encoding NMD and EJC components are conserved across metazoans, fungi, plants, and members of the unicellular supergroups: Amebozoa, Excavata, and SAR (stramenopiles, alveolates, Rhizaria) 72, 73. This leaves little doubt that the LECA had a functional version of this mRNA destabilization pathway. Although NMD is a uniquely eukaryotic process, careful bioinformatics analyses showed that a PIN domain present in a subset of endoribonuclease toxins participating in a bacterial post‐segregation cell‐killing program is conserved in some members of the Smg5‐Smg6‐Smg7 group 74, 75. Of these, metazoan Smg6 functions as an endoribonuclease 4 arguing that NMD might have evolved from an mRNA degradation system associated with a “biological conflict” in bacteria 38.

Interestingly, the evolutionary loss of the NMD/EJC genes observed in some species appears to correlate with a reduced incidence of introns in the corresponding genomes 72, 76. This argues that the evolution of introns in ancestral eukaryotes was possibly linked with the presence of the EJC‐dependent branch of NMD. At least two models rationalizing this relationship have been put forward. According to one of them, NMD evolved prior to expansion of retrotransposon‐like predecessors of spliceosomal introns in the eukaryotic genome and in fact facilitated this process by offsetting the penalty associated with splicing errors 76. An alternative model posits that NMD emerged following the main wave of intron expansion to protect the cell from incorrectly spliced transcripts 37.

Both models assume that the ancestral form of NMD was EJC‐dependent and that the EJC‐independent mechanisms appeared as its simplified derivatives in evolutionary branches affected by large‐scale elimination of introns. However, this scenario does not account for the fact that EJC‐independent NMD is known to occur in organisms with a normal complement of introns. Another limitation of the first model is that it does not specify selection forces that prompted eukaryotes to devise a sensor for exon‐exon junctions before retrotransposable introns became a serious problem. The second model explains the emergence of NMD as a part of the host defense against selfish genetic elements but implies that a multicomponent mRNA decay pathway was assembled virtually “from scratch” within a relatively short period of time.

## NMD might have emerged as a broad‐spectrum defense mechanism verifying translational authenticity of cellular transcripts

We believe that initial emergence of an EJC‐independent mechanism followed by subsequent acquisition of the EJC module might be a more plausible scenario. The early EJC‐independent version of NMD would have been immediately useful as a broad‐spectrum defense mechanism protecting the host cell against RNA viruses and retrotransposons unrelated to the group‐II self‐splicing introns. Differentiation between “self” and “nonself” in this system would rely on evaluation of mRNA translation patterns. A majority of cellular mRNAs escapes NMD, likely as a result of purifying selection against NMD‐promoting features. On the other hand, RNA pathogens must encode their replication and gene expression functions in a relatively small genome, limited by the capacity of the virion or/and error‐prone nature of RNA‐templated replication. This underlying requirement for genetic economy explains the abundance of virus‐ and retrotransposon‐encoded transcripts containing multiple open reading frames and other elements recognized by the NMD machinery.

Recent studies argue that NMD may be an important part of intrinsic immunity in extant eukaryotes. RNAi screens carried out by Balistreri and co‐workers identified Upf1, Smg5, and Smg7 as factors limiting replication of +RNA viruses from the *Togaviridae* family in mammalian cells 77. Genomic RNA in this group of viruses typically contains an upstream ORF encoding nonstructural proteins used for virus replication and a downstream ORF encoding structural components. The upstream ORF is translated directly from the full‐length genome, whereas ribosomal access to the downstream ORF requires production of a subgenomic mRNA lacking the upstream part. One of the consequences of this arrangement is that the downstream ORF becomes a part of a long 3′UTR during genomic RNA translation thus increasing the likelihood of NMD. Yet, removing the downstream ORF failed to alleviate the repressive effect of Upf1 on viral replication 77. Thus, other features of the viral genome may promote recruitment of the NMD machinery, and it will be important to identify such degradation determinants in the future.

Antiviral effects of NMD have been also shown in plants 78. In this case, Upf1, Upf3, and Smg7 (the only member of the Smg5‐Smg6‐Smg7 group conserved in *Arabidopsis*) inhibited replication of +RNA viruses from the families of *Alphaflexiviridae* and *Tombusviridae*. Similar to *Togaviridae*, these viruses use a subgenomic strategy to express a full complement of their proteins. This gives rise to unusually long 3′UTRs in some of the virus‐encoded RNAs. The authors showed that reducing the length of the 3′UTR in the corresponding RNA species rescued them from the inhibitory effect of NMD. Moreover, a +RNA virus from the *Potiviridae* family containing a single ORF and a relatively short 3′UTR escaped NMD restriction 78. Thus, the length of the 3′UTR is an important determinant allowing the plant NMD system to recognize a subset of RNA pathogens.

A study by Gloggnitzer et al. points at a wider role of NMD in the innate immunity in plants 79. The authors showed that Smg7 is required for regulation of expression levels of a subset of nucleotide‐binding leucine‐rich repeat receptors (NLR) involved in the host response to bacterial infection. Loss‐of‐function mutations in Arabidopsis *Smg7* and the *Upf1* genes lead to retarded development and seedling death as a result of elevated expression of antibacterial defense genes 80. Disruption of the disease resistance signaling is sufficient to rescue these autoimmunity‐related phenotypes 80. Interestingly, the activity of the NMD pathway naturally declines in plants infected by bacteria leading to increased expression of NMD‐targeted NLRs.

Upf1 has been additionally identified as a cell‐encoded protein interacting with bicistronic RNA of the human non‐LTR retroelement LINE‐1 81. Knocking down Upf1 led to a noticeable increase in the levels of the LINE‐encoded RNA and proteins, suggesting that Upf1 is a repressor of the retrotransposon‐specific gene expression program. However, this treatment also decreased the efficiency of LINE‐1 retrotransposition 81, a paradoxical result awaiting follow‐up analyses. In any case, this study confirms that NMD factors can mediate functional interaction between the host and the retrotransposon.

Of note, RBPs from the Staufen family can induce an NMD‐related process that destabilizes a subset of mammalian transcripts in a Upf1‐ and translation‐dependent manner 82. Staufen is recruited to specific 3′UTR sites containing intramolecular RNA hairpins, or more frequently, intermolecular duplexes formed by base‐pairing between complementary sequences originating from short interspersed repeats (SINEs), retroelements propagating using LINE‐encoded reverse transcriptase and endonuclease activities 32, 33, 34. It is conceivable that Staufen‐mediated decay emerged as an offshoot of NMD specializing in protection of the host cell from retrotransposon‐derived transcripts.

Viruses often evolve mechanisms allowing them to evade or disrupt host defenses and this trend is certainly apparent in the case of NMD. For example, Rous sarcoma retrovirus containing several ORFs in its genomic RNA contains a specialized stability element downstream of the first ORF encoding Gag and Pol proteins 83. This element allows the full‐length viral RNA to evade NMD by recruiting polypyrimidine‐tract binding protein (PTBP1/PTB), an abundant RBP in proliferating cells, which this virus prefers to infect 84. Another member of the *Retroviridae* family, human T‐lymphotropic virus type 1, inhibits the NMD machinery in part through interaction between Upf1 and the virus‐encoded Tax protein 85. Importantly, this stabilizes viral mRNAs in infected cells 85.

Similarly, coat protein of hepatitis C virus (HCV), a *Flaviviridae* family member, interferes with NMD by sequestering an EJC‐associated factor, PYM1/WIBG 86. The role of EJC‐dependent NMD in HCV biology is unclear since this virus replicates in the cytoplasm and does not encode spliceosomal introns. However, up‐regulation of cell‐encoded NMD targets may contribute to pathological effects associated with HCV infection 86. Finally, structural analysis of the nsp10 RNA helicase encoded by equine arteritis virus, a +RNA virus from the order of Nidovirales, uncovered a remarkable structural resemblance between this protein and Upf1 87. Although the significance of this finding still remains to be established, it is theoretically possible that nidoviruses employ this enzyme to interfere with the cellular NMD machinery 88.

## Tristetraprolin and related CCCH zinc‐finger proteins might have originated as a part of “nonself” RNA sequence recognition system

**Figure 3:**
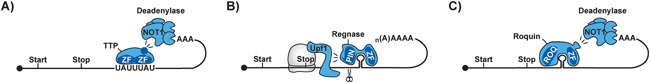
CCCH‐ZF‐RBP pathways destabilizing mRNA containing linear sequence motifs (TTP) or stem‐loop elements (Regnase and Roquin). **A:** Tristetraprolin (TTP)‐triggered degradation of mRNAs containing AU‐rich elements (AREs). A key step in this mechanism is recruitment of the Ccr4‐Caf‐Not mRNA deadenylation complex through interaction between TTP C‐terminal domain and Not1. **B:** Regnase‐mediated decay of mRNAs containing characteristic stem‐loop structures. This mechanism depends on Upf1 and the PIN‐domain endonuclease activity of the Regnase protein. **C:** Roquin targets secondary structure elements similar to those recognized by Regnase but destabilizes mRNA by recruiting the Ccr4‐Caf‐Not deadenylase.

Another characteristic form of controlled mRNA decay in eukaryotes relies on recognition of specific sequence motifs and structural elements present in a subset of cellular transcripts. RBPs containing CCCH‐type zinc fingers (ZFs) have been widely implicated in this regulation 7, 13, 89 (Fig. 3). As the name implies, this type of ZFs comprises three appropriately spaced cysteines followed by a single histidine residue.

One of the most extensively studied members of this protein group is mammalian Zfp36 also known as tristetraprolin (TTP) 7 (Fig. 3A). TTP, along with its paralogs Zfp36l1, Zfp36l2, and Zfp36l3, uses a centrally positioned tandem CCCH zinc finger (TZF) domain to bind unstructured AU‐rich RNA elements (AREs) typically containing one or several UAUUUAU consensus heptamers 7. These proteins additionally contain a C‐terminal domain that can interact with the Not1 subunit of the Ccr4‐Caf‐Not mRNA deadenylation complex 7. Consistent with this molecular feature, recruitment of TTP to its mRNA targets stimulates their deadenylation followed by decapping and Xrn1‐ and exosome‐dependent degradation 7.

TTP has been shown to regulate the stability of several mammalian mRNAs including those encoding proto‐oncogenes, growth factors, and cytokines (including some interferons) 7. Moreover, several brain‐enriched mRNAs containing UAUUUAU elements in their 3′‐untranslated regions (3′UTRs) are degraded in non‐neural cells expressing TTP at relatively high levels 90. TTP expression is naturally dampened during neural differentiation by miRNA miR‐9, thus promoting accumulation of these mRNAs and their protein products 90. Similarly TTP functions as a post‐transcriptional repressor of muscle stem cell differentiation by destabilizing mRNA of a pro‐myogenic factor, MyoD 91.

Conversely, basal levels of TTP can transiently increase in response to toll‐like receptor (TLR) and cytokine signaling 92. Since many proinflammatory mRNAs contain UAUUUAU motifs, this may function as a safety mechanism minimizing inflammation‐induced tissue damage. Consistent with this function, knockout mice lacking TTP develop severe autoimmunity‐related problems due to increased expression of one of its targets, mRNA of a potent mediator of inflammation, tumor necrosis factor (TNF) α (reviewed in 13).

Proteins containing the TTP‐like TZF domain have been identified in metazoa, fungi, plants, Amebozoa, and Excavata 93. Many of these proteins additionally contain the Not1‐interaction domain, which argues for conservation of their molecular functions. Indeed, the only *Drosophila* homolog of TTP called dTIS11 has been shown to destabilize ARE‐containing targets including mRNA of the antimicrobial peptide cecropin A1 94. Several TZF proteins have been shown to participate in stress and innate immunity responses in *Arabidopsis* 89. The fission yeast TTP homolog Zfs1 is known to regulate stability of several mRNAs encoding cell‐cell adhesion proteins, and its genetic inactivation leads to increased cell clustering, or flocculation 95. Flocculation is considered to be a form of stress response in yeasts protecting the inner cells of the flocs against environmental challenges. Despite the vast evolutionary distance separating these species, the TZF domain of Zfs1 is functionally interchangeable with those of its homologs of mammalian, insect, plant, and fungal origins 95. Taken together, these data indicate that TTP‐like proteins likely evolved prior to radiation of the main eukaryotic supergroups and rapidly assumed regulatory roles related to cellular stress response. Given the prevalence of innate immunity‐ and inflammation‐related targets regulated by these proteins in contemporary metazoans, one can speculate that ancestral forms of TTP were related to cellular defense against RNA pathogens.

In line with this model, a large fraction of human ARE sequences is associated with Alu elements, an abundant SINE group 96. SINEs require a 3′‐terminal polyA tail for retrotransposition, which results in the appearance of U‐rich sequences when a SINE copy is inserted into a host gene in a reverse orientation. These may function as TTP sites, especially after acquiring a few U‐to‐A transversions 96. Since poly(A) sequences are also required for mobility of LINEs and other types of non‐LTR retrotransposons 33, 34, it is conceivable that TTP‐like proteins participated in surveillance of the LECA transcriptome for defects associated with retrotransposon activity. Interestingly, other ARE‐specific regulators including AUF1/hnRNP D and KHSRP are also conserved across eukaryotic supergroups (97, 98; and our blastp results) and might have contributed to this primeval defense mechanism.

## Regnase and roquin protein families might have evolved to detect “nonself” RNA structures

Liang et al. provide an important insight into the CCCH ZF protein evolution 99. The authors compared sequences of 58 such proteins encoded in the mouse genome and identified Zc3h12 and Rc3h among the closest relatives of the TTP family (see Fig. 1 in 99). Zc3h12 is represented by four (Zc3h12a‐d) and Rc3h by two paralogs (Rc3h1/Roquin1 and Rc3h2/Roquin2) in the mouse genome. Similar to TTP and its paralogs, these proteins containing a single CCCH zinc finger domain participate in regulated mRNA destabilization (see below).

The Zc3h12 ZFs are especially closely related to the TTP ZFs (see Fig. 3 in 99). The best‐studied member of this family is Zc3h12a, also know as MCPIP1 or Regnase (Fig. 3B). It is known to destabilize a subset of mRNAs including those of pro‐inflammatory cytokines IL6 and IL12b and IL1b and factors involved in T cell activation (e.g. Icos, c‐Rel, and Ox40) 100, 101, 102. Besides its ZF, Regnase contains a catalytically active PIN domain mediating mRNA degradation. As mentioned above, a similar domain is also present in the NMD endoribonuclease Smg6, arguing for a distant phylogenetic relationship between the two degradation systems. Notably, Regnase has indeed been shown to destabilize its targets in a Upf1‐dependent manner 103.

Regnase recognizes secondary structure elements containing a stem and a trinucleotide loop and its PIN domain participates in RNA binding along with the ZF 103, 104. Interestingly, Roquin1 and Roquin2 proteins recognize an overlapping set of structural elements and promote destabilization of their mRNA targets by recruiting the Ccr4‐Caf‐Not deadenylase complex 103, 105 (Fig. 3C). RNA binding of Roquins depends on a conserved ROQ domain; however, the ZF element is also required at least for interaction with stem‐loops enriched in U 105, 106. Notably, the systemic immunity phenotypes of mice lacking functional *Zc3h12a/Regnase* or *Rc3h1/Roquin1* genes are somewhat evocative of the effects brought about by the *Zfp36/TTP* knockout 13. These phylogenetic and functional similarities among the three protein families argue that the corresponding mRNA decay mechanisms might have emerged from common evolutionary roots.

Importantly, several recent studies suggest that, in addition to its role in cellular mRNA metabolism, Regnase can restrict replication of +RNA viruses from the families of *Flaviviridae* (HCV, Japanese encephalitis virus, and dengue virus), *Picornaviridae* (Encephalomyocarditis virus) and *Togaviridae* (Sindbis virus). It may also interfere with replication of some −RNA viruses (Influenza A virus; *Orthomyxoviridae*) and reverse‐transcribing lentiviruses (Human and Simian immunodeficiency viruses; *Retroviridae*) 107, 108, 109, 110. These activities appear to involve destabilization of viral RNAs in a manner requiring functional PIN and ZF domains. At least in the case of *Flaviviridae*, the ZF domain was also shown to stimulate binding of Regnase to viral RNAs 108, 109. Consistent with its antiviral function, Regnase expression has been shown to increase in response to infection and TLR signaling 109, 111.

Although cis‐elements mediating antiviral effects of Regnase are presently unknown, efficient replication of RNA viruses often requires conserved RNA structures that might, at least in theory, function as a Regnase “specificity code.” Interestingly, repression of viral replication by Regnase depends on the ability of this protein to form oligomers 104. It is plausible that each of these oligomers may simultaneously recognize several stem‐loop elements in a single viral RNA. A conceptually similar multipoint interaction with a complex tertiary RNA structures has been proposed for Zc3hav1/ZAP 112, an interferon‐inducible factor containing four CCCH ZFs and participating in antiviral defense as well as regulation of some cellular transcripts 113, 114, 115.

All in all, it is tempting to speculate that CCCH‐ZF RBPs are extant descendants of an ancient immunity mechanism interfering with RNA pathogen replication in eukaryotic cells. Duplicating the ZF unit within a single polypeptide (as occurred in TTP and Zc3hav1), combining it with other RNA interaction domains (as in Regnase and Roquins) or forming quaternary protein units (as in Regnase and ZAP) might have extended versatility of this system allowing it to recognize a wide range of linear and folded RNA epitopes. Interestingly, no prokaryotic proteins containing more than one CCCH ZF have been identified so far (93; and our blastp results). On the other hand, some dsDNA viruses from the *Iridoviridae* family and +RNA viruses from the order of Nidovirales (mentioned in the previous chapter as viruses with a Upf1‐like RNA helicase) encode TZF‐domain proteins of unknown function (Table S1, Supporting Information; also see 93). This might be a result of horizontal transfer of TZF sequences between viral and cellular genomes arguing for a special role of this protein module in host‐pathogen interactions.

## Conclusions

In summary, several lines of evidence point at possible emergence of NMD and CCCH‐ZF RBP‐mediated mRNA destabilization mechanisms as intrinsic immunity systems. Similar to the RNAi pathway frequently mentioned in this context, origins of these mechanisms likely coincided with rampant expansion of RNA viruses and retroelements. By recognizing mRNA translation patterns and detecting linear and structured RNA epitopes, these evolutionary innovations might have substantially improved the ability of the host to discriminate between cellular “self” and viral “nonself.”

This scenario implies that the NMD‐ and the CCCH‐ZF‐RBP‐specific features are continuously depleted from cell‐encoded transcripts by purifying selection. On the other hand, considerations of genetic economy and replication efficiency maintain these features in RNA pathogens at a steady level. The advent of “professional” immunity systems might have allowed repurposing of these post‐transcriptional mechanisms for mRNA quality control and gene regulation in the host cell. This exaptation was clearly only partial since NMD and CCCH‐ZF RBPs retain some antiviral and immunomodulatory functions in the present‐day eukaryotes.

The multifaceted and highly intertwined relationship between cellular and viral RNA metabolisms 116, 117, 118 indicates that other post‐transcriptional mechanisms limiting stability, processing, and translational efficiency of eukaryotic RNA transcripts might have evolved via similar routes. Therefore, one should anticipate a wider range of mRNA degradation processes to be uncovered in future screens for intrinsic antiviral factors. One important prediction of our hypothesis is that these novel post‐transcriptional components should be especially abundant in simple organisms lacking PRR‐based and adaptive branches of immunity but susceptible to RNA pathogen infections.

## Supporting information

As a service to our authors and readers, this journal provides supporting information supplied by the authors. Such materials are peer reviewed and may be re‐organized for online delivery, but are not copy‐edited or typeset. Technical support issues arising from supporting information (other than missing files) should be addressed to the authors.

**Table S1**. Viral TZF‐containing proteins showing detectable sequence similarity to human TTP/ZFP36 (blastp, May 2016)
