# Supplementary material for: Exaptive origins of regulated mRNA decay in eukaryotes
Source: Bioessays. 2016 Jul 20;38(9):830–8. doi: 10.1002/bies.201600100 (PMC5031192; doi:10.1002/bies.201600100)
Supplement: Supplementary file 1 — Table S1. Viral TZF‐containing proteins showing detectable sequence similarity to human TTP/ZFP36 (blastp, May 2016) [file BIES-38-830-s001.pdf]

Table S1. Viral TZF-containing proteins showing detectable sequence similarity to human TTP/ZFP36 (blastp, May 2016)

Virus	Type of genome	Taxon	Protein	Identity	E value	Accession
Scale drop disease virus	dsDNA	*Iridoviridae*	ORF_096L	47%	7.00E-12	YP_009163857.1
Ball python nidovirus	+RNA	Nidovirales	pp1ab replicase polyprotein	34%	8.00E-04	YP_009052475.1
Ball python nidovirus	+RNA	Nidovirales	pp1a replicase polyprotein	31%	0.001	YP_009052476.1
Python nidovirus	+RNA	Nidovirales	pp1a replicase polyprotein	33%	0.002	AII00824.1
Lymphocystis disease virus 1	dsDNA	*Iridoviridae*	Tristetraprolin-like zinc finger protein C3H	29%	0.049	NP_078696.1
